# Editorial: Plant-derived natural compounds in drug discovery: The prism perspective between plant phylogeny, chemical composition, and medicinal efficacy

**DOI:** 10.3389/fpls.2022.1042695

**Published:** 2022-10-07

**Authors:** Da-Cheng Hao, Chun-Nian He, Richard W. Spjut, Pei-Gen Xiao

**Affiliations:** ^1^ School of Environment and Chemical Engineering, Biotechnology Institute, Dalian Jiaotong University, Dalian, China; ^2^ Institute of Molecular Plant Science, University of Edinburgh, Edinburgh, United Kingdom; ^3^ Institute of Medicinal Plant Development, Chinese Academy of Medical Sciences and Peking Union Medical College, Beijing, China; ^4^ World Botanical Associates, Bakersfield, CA, United States

**Keywords:** pharmacophylogeny, plant phylogeny/phylogenomics, chemical composition, biological activity, multi-omics

Plant based natural medicine research has evolved from a long history going back to the beginning of human civilization to the present in building on innovative research method systems of pharmacophylogeny and pharmacophylogenomics (Hao and Xiao, 2020). “Pharmacophylogeny” was conceived by Professor Xiao Pei-Gen four decades ago as a result of long-term studies of Chinese researchers, especially since the 1950s. The objective is to disentangle the intricate relationships and connectivity between medicinal plant phylogeny, phytochemical profiles and bioactivities/therapeutic utilities (**Figure 1**), so as to benefit pioneering plant-based drug research and development (R&D). The historical research in this field while continuing with the status quo, pharmacophylogeny, has become increasingly familiar to more and more researchers. “Pharmacophylogenomics” is proposed to reflect the mounting range of applications of omics based pharmacophylogeny in phytomedicine research. Pharmacophylogeny/pharmacophylogenomics is a multidisciplinary integration, involving molecular phylogeny/phylogenomics, plant morphology, chemotaxonomy, phytochemistry, molecular biology and omics, ethnopharmacology/pharmacology, and the like; for example, Spjut (1985) had recognized the genus as the lowest chemotaxonomic level of diversity to suggest a phytogeographical approach in searching novel antitumor compounds. In the advent of phylogeny, this could become a phylogeographic approach. Pharmacophylogeny suggests that healing plants of the related taxonomic groups are more likely to possess the analogous chemical profiles/efficacies (**Figure 1**), which is a law explored from long-term herbal medicine practice, and is used in practice after being confirmed and perfected by scientific research. It played an active role in bioprospecting domestic resources to replace imported medicines. Currently, it is very useful to expand medicinal plant resources (Cui et al.), along with authentication/quality regulation of herbal medicines, and predicting the chemicals or bioactive constituents of herbals and identification/quantification of chemicals (**Figure 1**). In the coming years, pharmacophylogeny and pharmacophylogenomics could be more powerful in mining original natural medicines, refining ethnopharmacology understandings, therefore advancing the workable protection and application of old/natural remedial resources.

**Figure 1:**
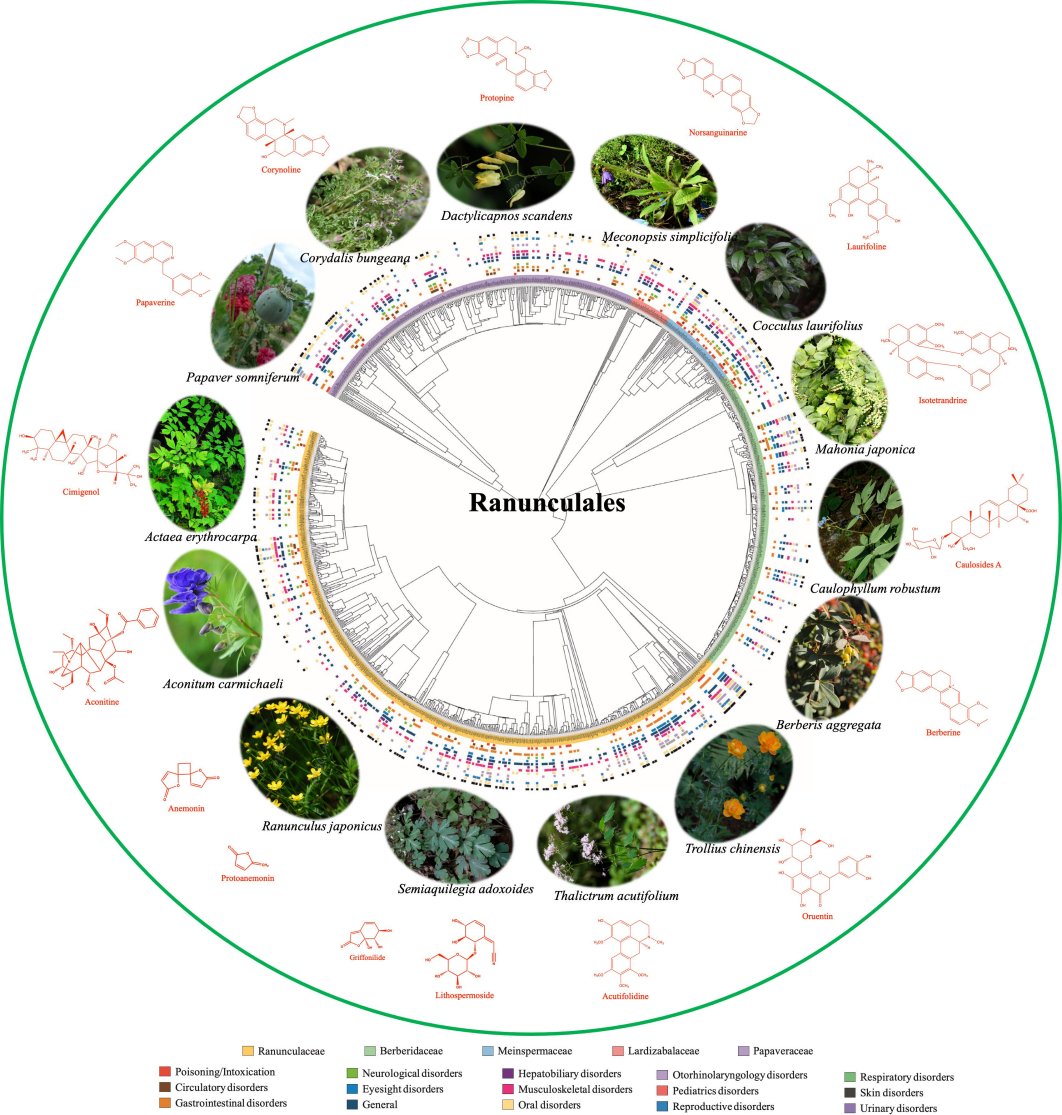
An illustrated overview of pharmacophylogeny, taking the order Ranunculales as an example.

The goal of this Research Topic is to gain novel mechanism insights into the phylogeny/evolution, phytometabolites and pharmacological effects of selected medicinal genera/families. Where possible, we propose that such explorations should be conducted within the context of pharmacophylogeny and/or pharmacophylogenomics. To this end, this Research Topic presents three original articles that reconstructed the phylogenetic trees of *Scutellaria* (Shen et al.), tribe Polygonateae (Wang et al.) and *Arnebia* (Sun et al.) respectively based on the whole chloroplast (cp) genome sequences. The Lamiaceae genus *Scutellaria* has more than 360 species, among which over 70 species have a long history of medicinal use. The molecular phylogeny and metabolomic information are essential to understand the medicinal value of this genus and to develop alternative medicinal resources. The complete cp genomes of 17 *Scutellaria* species, which were used to reconstruct the phylogenetic tree (Shen et al.), disagrees with the traditional morphological grouping. Convincingly, *S. baicalensis* (*Radix Scutellariae* in traditional Chinese medicine (TCM)) is most closely related to *S. viscidula*, followed by *S. hypericifolia*, *S. amoena* and *S. likiangensis*; correspondingly, the metabolomic analyses and phytometabolite content determination reveal the overall similarity of phytometabolite profiles between *S. baicalensis* and its four substitute species. The cp genome sequencing also suggests petA-psbL as a potential barcoding marker for distinguishing *S. baicalensis* and its substitutes, and the interspecific chemodiversity could lead to development of novel clinical utility. A set of cp genome sequences of Asparagaceae tribe Polygonateae (Wang et al.) is essential for unearthing the links between phylogenomics and chemotaxonomy of medicinal taxa therein. The complete cp genomes of 26 Polygonateae species were *de novo* assembled and characterized; the cp genome-based phylogeny suggests the monophyly of *Polygonatum*, an important TCM genus, and also suggests that *Heteropolygonatum* may be its sister group, except that *Disporopsis*, *Maianthemum* and *Disporum* may have diverged earlier. On the contrary, the phyllotaxy of *Polygonatum* is not stable at the intraspecies level, which cannot be taxonomically used as the unique morphology marker.

*Arnebiae Radix* is an old oriental medication with diverse activities. The genome skimming methods were utilized to obtain cp genomes of five *Arnebia* species (Sun et al.), by which the phylogenetic relationship of five *Arnebia* species was completely resolved. The origin plants of *Arnebiae Radix A. guttata* and *A. euchroma* were of high genetic diversity, and had three and two subclades respectively. The cp genome is an useful genetic resource for phylogeny and evolution studies at both species and subspecies/population levels. The genetic resources provided in these studies will aid the conservation and exploitation of various medicinal taxonomic groups. Notwithstanding, hybridization has led to incomplete lineage sorting and polyploidy during long-term evolution of taxonomic groups; cp phylogeny does not equal to the exact species one (Lu et al., 2022). In RNA-Seq based phylotranscriptomics, nuclear orthologous genes are concatenated to reconstruct the phylogenetic tree (Hao et al., 2021), which, along with genome skimming (Sun et al.) and Hyb-Seq (Lu et al., 2022), represents the promising complementary approach in pharmacophylogenomics.

According to pharmacophylogeny, taxa in sister phylogeny groups have closely related genetic features (Hao and Xiao, 2020). Thus, they are more likely to possess analogous biosynthesis pathways and their chemical arsenals could be more similar (Liu et al., 2021). The various tiers of chemical similarity result in the global resemblance of bioactivity or pharmacological efficacy (Hao et al., 2022a; 2022b Liu et al.). Pharmacophylogeny successfully guides the development of novel curative taxa (Hao and Xiao, 2020), while circumventing the limitations of old-style approaches and enabling the targeted studies. In contrast, the random discovery (accidental discovery) relies too much on luck; new molecular entities obtained by combinatorial chemistry, high throughput screening or computer aided drug design very often fail due to improper pharmacokinetic and pharmacodynamic properties (Hao et al., 2022c). A complete knowledgebase of chemodiversity/phytometabolism of systematic groups of interest is an essential base for pharmacophylogeny. This Research Topic presents a few original articles that carefully investigate the specialized metabolism and chemodiversity of *Chrysanthemum* (Hao et al.), *Oxytropis* (Jia et al.), *Broussonetia* (Jiao et al.), *Schisandra* (Liu et al.), *Bletilla* (Liu et al.) and *Verbena* (Peng et al.). More than 9,000 flavonoids are distributed in 245 families of seed plant (Zhang et al., 2020). In *Broussonetia papyrifera* leaves, flavonoids accumulate gradually in leaf development to until its maturity (Jiao et al.), and female plants have greater flavonoid leaf content than in male plant, despite their analogous composition. The 192 identified flavonoids include flavonols, flavones, flavan-3-ols, flavonoid carbonoside, anthocyanins, among others, and their biosynthesis follows the well-known flavonoid biosynthetic pathways. Some differentially expressed genes and metabolites along the flavonoid biosynthetic pathway were quantified by transcriptome sequencing and metabolomic analyses respectively.

The phenolic acids are distributed in 167 families of seed plant (Zhang et al., 2020). Natural phenolic compounds are commonly distributed in food and TCM (Hao et al.). In a suspension culture of *Bletilla striata* (Liu et al.), the content of Dactylorhin A peaked at the earliest, i.e., nine days post inoculation, followed by p-hydroxybenzylalcohol, Militarine and Coelonin. Based on full-length transcriptome data, multiple unigenes involved in the biosynthesis of HBA, militarine, dactylorhin A and coelonin were identified. In the transcriptome analysis of *Verbena officinalis*, 206, 229 and 115 unigenes were identified in the biosynthetic pathways of iridoid glycoside, phenylethanol glycoside and flavonoid respectively (Peng et al.). The contents of these phytometabolites were highest in leaves, followed by stem and root, which is consistent with inter-tissue variations of biosynthetic gene expression levels.

The above studies not only contribute a large number of unigenes for the ortholog extraction and phylotranscriptomic inference, but also greatly enrich the phytometabolite database at the genus/species level. In light of the expanding knowledge of plant chemodiversity, it is possible to study the phylogenetic distribution of diverse types of compounds, e.g., less studied pyrone glycoside (Shen et al.), at the subfamily, tribe, genus or subgenus levels, so as to bioprospecting medicinal taxa more precisely.

A triple helix systems perspective, i.e., an essentially holistic view, would be established if we qualitatively and quantitatively investigate the association between molecular phylogeny and phytochemical data (Zhang et al., 2020), between phylogeny and bioactivity/efficacy (Hao et al., 2022a; 2022b), and between phytometabolite and bioactivity (Song et al.; Sun et al.). Based on extensive ethnomedicinal uses, the thorough phytochemistry and bioactivity characterizations contribute to the safe administration of herbals and discovery of lead compounds (Souza et al., 2018). In total, 167 compounds were identified from essential oils (EOs) of six Lamiaceae herbs by GC-MS analysis (Sun et al.). All EOs had promising anti-inflammatory activity in rats with adjuvant arthritis, and *Perilla frutescens* EO was the best. They alleviated the joint swelling in the rat models; the lymphocyte infiltration and cartilage damage were significantly inhibited. The TNF-α, IL-1, and IL-6 were reduced in rat articular tissues by EOs. Six EOs had substantial toxicity on tumor cell lines; they also had the significant antioxidant activity, and *Salvia japonica* EO was the strongest. Berberine, baicalin and saponin from *Coptis chinensis*, *Scutellaria baicalensis* and *Vincetoxicum atratum* respectively were combined to produce the formula BBS (Song et al.), and effects of BBS on tumor growth and protein expression were as strong as those of formula HHB, a combination of the above three herbs. Although these bottom-up and intermediate approaches cannot replace the top-down approach, i.e., treatment investigations of clinical patients, they are very important in confirming or falsifying tentative conclusions obtained from the phylogenetic signals in phytometabolites or bioactivity/efficacy across species phylogenetic tree. The bioactivity studies provide more reference data for pharmacophylogeny research and drug discovery from ethnomedicinal plants.

In summary, the articles of this Research Topic illustrate the newest knowledge and prospects of pharmacophylogeny, pharmacophylogenomics and related concepts, and their mounting usefulness in phytomedicine R&D. The deeper investigations of genomics of curative plants, metabolomics, and ethnopharmacology-based bioactivity will enable the workable protection and consumption of botanical possessions. Of note, species relationships may be cryptic due to genetic and/or epigenetic factors, or species of the same taxonomic group may be morphologically and biochemically distinct, making it possible for closely related taxa to have diametrically opposite clinical effects. During long-term evolution, the phytochemical diversification parallels the expansion of biological complexity. Biologically active compounds are generally specialized metabolites, and the distribution of these compounds on phylogenetic trees of different levels is very worthy of deeper study. Many medicinally important phytometabolites, e.g., alkaloids, terpenoids and phenylpropanoids, can be further subdivided structurally, and the phylogenetic signals of different subtypes/subclasses could be distinct. For compounds with similar structures, not only the resemblances and variances of their bioactivities should be explored, but also those of their pharmacokinetic and pharmacodynamic properties should be clarified one by one (Hao et al., 2022c). Therefore, there is a great deal of work to be conducted on various topics of pharmacophylogeny. During the past two decades, various omics techniques have continued to evolve towards elucidating the cryptic connections between plant phylogeny, phytometabolites and their biosynthesis, as well as their bioactivities, which promisingly provide lead entities to develop novel plant-derived drugs.

## Author contributions

D-CH and C-NH prepared the draft and finalized the manuscript. P-GX and RS edited and corrected the draft. All authors contributed to the article and approved the submitted version.

## Funding

The authors are grateful to the Journal for presenting this Editorial article. D-CH is supported by China Scholarship Council (202108210156). This work is also funded by the Innovation Team and Talents Cultivation Program of the National Administration of Traditional Chinese Medicine (ZYYCXTDD-202005) and CAMS Innovation Fund for Medical Sciences (CIFMS; 2021-I2M-1–071).

## Conflict of interest

The authors declare that the research was conducted in the absence of any commercial or financial relationships that could be construed as a potential conflict of interest.

## Publisher’s note

All claims expressed in this article are solely those of the authors and do not necessarily represent those of their affiliated organizations, or those of the publisher, the editors and the reviewers. Any product that may be evaluated in this article, or claim that may be made by its manufacturer, is not guaranteed or endorsed by the publisher.
